# Single-cell transcriptomics reveal different maturation stages and sublineage commitment of human thymic invariant natural killer T cells

**DOI:** 10.1093/jleuko/qiad113

**Published:** 2023-09-23

**Authors:** Kristina Maas-Bauer, Natalie Köhler, Anna-Verena Stell, Melissa Zwick, Swati Acharya, Anne Rensing-Ehl, Christoph König, Johannes Kroll, Jeanette Baker, Stefanie Koßmann, Amandine Pradier, Sisi Wang, Mylène Docquier, David B Lewis, Robert S Negrin, Federico Simonetta

**Affiliations:** Division of Blood and Marrow Transplantation and Cellular Therapy, Stanford University, Center for Clinical Sciences Research Building, 269 W. Campus Drive, Stanford, CA 94305, United States; Department of Hematology, Oncology, and Stem Cell Transplantation, Medical Center—University of Freiburg, Faculty of Medicine, Hugstetter Str. 55, Freiburg 79106, Germany; Department of Hematology, Oncology, and Stem Cell Transplantation, Medical Center—University of Freiburg, Faculty of Medicine, Hugstetter Str. 55, Freiburg 79106, Germany; CIBSS—Centre for Integrative Biological Signalling Studies, University of Freiburg, Schänzlestr. 18, Freiburg 79104, Germany; Department of Hematology, Oncology, and Stem Cell Transplantation, Medical Center—University of Freiburg, Faculty of Medicine, Hugstetter Str. 55, Freiburg 79106, Germany; Department of Hematology, Oncology, and Stem Cell Transplantation, Medical Center—University of Freiburg, Faculty of Medicine, Hugstetter Str. 55, Freiburg 79106, Germany; Sean N. Parker Center for Asthma and Allergy Research, Department of Medicine, Stanford University, 240 Pasteur Dr, Stanford, CA 94304, United States; Institute for Immunodeficiency, Center for Chronic Immunodeficiency, Medical Center-University of Freiburg, Faculty of Medicine, Breisacher Str. 115, Freiburg 79106, Germany; Institute for Immunodeficiency, Center for Chronic Immunodeficiency, Medical Center-University of Freiburg, Faculty of Medicine, Breisacher Str. 115, Freiburg 79106, Germany; Faculty of Biology, University of Freiburg, Schänzlestr. 1, Freiburg 79104, Germany; Department of Cardiovascular Surgery, Heart Center Freiburg University, Hugstetter Straße 55, Freiburg 79106, Germany; Division of Blood and Marrow Transplantation and Cellular Therapy, Stanford University, Center for Clinical Sciences Research Building, 269 W. Campus Drive, Stanford, CA 94305, United States; Department of Hematology, Oncology, and Stem Cell Transplantation, Medical Center—University of Freiburg, Faculty of Medicine, Hugstetter Str. 55, Freiburg 79106, Germany; Division of Hematology, Department of Oncology, Geneva University Hospitals, Rue Gabrielle-Perret-Gentil 4, Geneva 1205, Switzerland; Translational Research Center for Oncohematology, Department of Medicine, Faculty of Medicine, University of Geneva, Rue Michel-Servet 1, Geneva 1211, Switzerland; Translational Research Center for Oncohematology, Department of Medicine, Faculty of Medicine, University of Geneva, Rue Michel-Servet 1, Geneva 1211, Switzerland; iGE3 Genomics Platform, University of Geneva, Rue Michel-Servet 1, Geneva 1211, Switzerland; Department of Genetics & Evolution, University of Geneva, Rue Michel-Servet 1, Geneva 1211, Switzerland; Division of Allergy, Immunology and Rheumatology, Department of Pediatrics, Stanford University School of Medicine, 240 Pasteur Dr, Stanford, CA 94304, United States; Division of Blood and Marrow Transplantation and Cellular Therapy, Stanford University, Center for Clinical Sciences Research Building, 269 W. Campus Drive, Stanford, CA 94305, United States; Division of Blood and Marrow Transplantation and Cellular Therapy, Stanford University, Center for Clinical Sciences Research Building, 269 W. Campus Drive, Stanford, CA 94305, United States; Division of Hematology, Department of Oncology, Geneva University Hospitals, Rue Gabrielle-Perret-Gentil 4, Geneva 1205, Switzerland; Translational Research Center for Oncohematology, Department of Medicine, Faculty of Medicine, University of Geneva, Rue Michel-Servet 1, Geneva 1211, Switzerland

**Keywords:** differentiation, human invariant natural killer T cells, iNKT, iNKT subsets, scRNAseq, thymic development

## Abstract

Invariant natural killer T cells are a rare, heterogeneous T-cell subset with cytotoxic and immunomodulatory properties. During thymic development, murine invariant natural killer T cells go through different maturation stages differentiating into distinct sublineages, namely, invariant natural killer T1, 2, and 17 cells. Recent reports indicate that invariant natural killer T2 cells display immature properties and give rise to other subsets, whereas invariant natural killer T1 cells seem to be terminally differentiated. Whether human invariant natural killer T cells follow a similar differentiation model is still unknown. To define the maturation stages and assess the sublineage commitment of human invariant natural killer T cells during thymic development, in this study, we performed single-cell RNA sequencing analysis on human Vα24^+^Vβ11^+^ invariant natural killer T cells isolated from thymocytes. We show that these invariant natural killer T cells displayed heterogeneity, and our unsupervised analysis identified 5 clusters representing different maturation stages, from an immature profile with high expression of genes important for invariant natural killer T cell development and proliferation to a mature, fully differentiated profile with high levels of cytotoxic effector molecules. Evaluation of expression of sublineage-defining gene sets revealed mainly cells with an invariant natural killer T2 signature in the most immature cluster, whereas the more differentiated ones displayed an invariant natural killer T1 signature. Combined analysis with a publicly available single-cell RNA sequencing data set of human invariant natural killer T cells from peripheral blood suggested that the 2 main subsets exist both in thymus and in the periphery, while a third more immature one was restricted to the thymus. Our data point to the existence of different maturation stages of human thymic invariant natural killer T cells and provide evidence for sublineage commitment of invariant natural killer T cells in the human thymus.

## Teaser

Human thymus contains different maturation stages of invariant natural killer T cells and facilitates sublineage commitment with invariant natural killer T2 properties in the most immature cells and an invariant natural killer T1 signature in the more differentiated clusters.

Human thymus contains different maturation stages and facilitates sublineage commitment of invariant natural killer T cells.

## 1. Introduction

Invariant natural killer T (iNKT) cells are a rare subset of innate lymphocytes representing less than 1% of the total lymphocyte population in both humans and mice. iNKT cells express a semi-invariant TCR, consisting of a Vα14Jα18 chain paired with a limited selection of beta chains in mice and Vα24Jα18 typically pairing with Vβ11 in humans, which recognize glycolipids presented in the context of the nonpolymorphic, histocompatibility complex (MHC)-like molecule CD1d.^1,2^ Upon stimulation, iNKT cells can promptly release a wide range of cytokines, allowing iNKT cells to exert a spectrum of pleiotropic functions, ranging from antitumor effects to immune-regulatory activity.^3,4^

Traditionally, thymic development of murine iNKT cells has been defined by different maturation stages based on the surface expression of CD24, CD44, and NK1.1: CD24^hi^CD44^lo^, NK1.1^lo^ immature precursor iNKT cells (iNKT0 or iNKTp)^5^; CD24^lo^CD44^lo^, NK1.1^lo^ stage 1 iNKT cells; CD24^lo^CD44^hi^, NK1.1^lo^ stage 2 iNKT cells; and CD24^lo^CD44^hi^, NK1.1^hi^ stage 3 iNKT cells. Interestingly, these maturation stages have been associated with different functional properties, with stage 1 and 2 iNKT cells producing interleukin (IL) 4 and IL-10 and stage 3 iNKT cells producing interferon-γ, with limited proliferation potential compared to stage 1 and 2 iNKT cells.^6–8^ Such a model has been challenged by recent reports based on single-cell genomic analyses identifying the coexistence of different maturation iNKT stages in the murine thymus and also suggesting that populations with mixed characteristics exist.^9^

Partly opposed to the theory of different maturation stages, several studies indicated the existence of at least 3 terminally differentiated, murine iNKT sublineages, namely, Th-1–like iNKT (iNKT1), Th-2–like iNKT (iNKT2), and Th-17–like iNKT (iNKT17) cells.^10,11^ These subsets are characterized by the differential expression of the transcription factors promyelocytic leukemia zinc finger (PLZF), GATA binding protein 3 (GATA3), and T-bet and RAR-related orphan receptor gamma (RORγt) (iNKT1: PLZF^lo^, T-bet^+^, iNKT2: PLZF^hi^ GATA3^hi^, iNKT17: PLZF^int^RORγt^+^)^10,11^; produce a unique cytokine profile^10^; and have special distribution in tissues.^12^ We recently demonstrated that distinct iNKT sublineages exert different functions, with iNKT2 and iNKT17 displaying immunoregulatory properties while iNKT1 exerts the strongest cytotoxic activity.^13^ Importantly, in contrast to what is observed in conventional T cells whose Th1, Th2, or Th17 lineage commitment takes place upon antigen encounter in the periphery, iNKT cell sublineage differentiation already takes place at the thymic level.^10,11^ Importantly, a recent single-cell report on thymic iNKT cells of prepubertal pigs showed big differences between murine and porcine iNKT cells.^14^ In this study, porcine iNKT cells were unexpectedly homogeneous, with 97% of cells expressing an iNKT2 genotype.

Despite the progress in understanding iNKT cell development and differentiation in animals, little is still known about human iNKT cell thymic development. Early studies studying human iNKT cell heterogeneity mainly focused on the analysis of peripheral blood.^15,16^ Studies investigating maturation processes of human iNKT cells at the thymic level based on such phenotypic heterogeneity have shown that, similarly to murine iNKT cells, the predominant iNKT cell population in neonatal human thymus includes CD4^+^CD161^−^ iNKT cells, whereas CD4^−^CD161^+^ iNKT cells accumulate with age.^17,18^ Whether these subsets of human thymic iNKT cells correspond to distinct maturation stages is still unclear. Moreover, it is still unknown whether human iNKT cells commit at the thymic level toward any of the sublineage profiles reported in mice.

## 2. Methods

### 2.1 Human samples

Thymi were obtained after being removed from 5 newborn/infant donors undergoing cardiac surgery between 5 d and 13 mo of age for cardiac diseases without evidence of immunologic diseases. The analysis of human thymus samples obtained as surgical waste was reviewed and approved by Stanford University, Institutional Review Board (IRB Protocol #16877; directors Drs. David B. Lewis and Swati Acharya, Pediatric Cardiovascular Surgery, Stanford Children's Health) and the Ethics Committee of the University of Freiburg (CardioVascular BioBank approval #393/16, study protocol #10/20). Single-cell suspensions were obtained by mechanical dissociation, washed, cryopreserved in fetal calf serum and 10% DMSO, and stored in liquid nitrogen until use.

### 2.2 Preparation of thymic iNKT cells

Thymocytes were thawed at 37 °C in RPMI 1640 containing fetal calf serum (FCS) 30%, penicillin-streptomycin 1%, DNase I (10 μg/mL; Sigma), and heparin 20 U/mL. After washing, fluorescence-activated cell sorting (FACS) surface staining was performed on ice, after FC receptor blockage, using the following antibodies: Vα24 (clone C15, FITC, Beckman Coulter), Vβ11 (clone C21, APC, Beckman Coulter), CD3 (BV605, Biolegend), CD14 (APC Cy7, Biolegend or PE Cy7, Biolegend), CD19 (APC Cy7, Biolegend), and LIVE/DEAD Fixable Aqua Dead Cell Stain Kit (Invitrogen). CD3^+^, Vα24^+^, Vβ11^+^, CD14^−^, and CD19^−^ live cells were FACS-sorted on a FACS Aria-III (BD Biosciences).

### 2.3 Single-cell RNA sequencing

Single-cell libraries were generated separately from FACS-sorted human thymic iNKT cells using the Chromium Controller Single-Cell Instrument and Chromium Single Cell 3′ Library & Gel Bead Kit v2 (donors 1, 2, and 3) and Chromium X instrument and Chromium Single Cell 3′ Library & Gel Bead Kit v3 (donors 4 and 5) (10× Genomics). Sample demultiplexing, barcode processing, alignment to the GRCh38 assembly of the human genome, and single-cell 3′ gene counting were performed using the Cell Ranger Suite versions 2.1 and 6.1.2. A total of 1,217 cells were sequenced, with 404,267 mean reads per cell and 1,250 (984 to 1,949) median genes per cell. The detailed metrics per sample are provided in Supplementary Table 1. Cells with unique gene counts <100 or >2,500, as well as cells with >10% of mitochondrial genes, were excluded from the analysis. Single-cell RNA sequencing (scRNA-seq) analysis was performed using the Seurat R package, version 4. Data integration was performed using the IntegrateData() function after identifying anchors using the FindIntegrationAnchors() function. Pseudotime analysis was performed using the Monocle3 package. iNKT sublineage-specific gene signatures were generated on murine iNKT in our previous work^13^ or extracted from the original description by Engel et al.^11^ Human orthologs were identified using the biomaRt version 2.46.3 package. The composite expression score for each iNKT sublineage gene signature was calculated using Seurat's AddModuleScore function. Previously published scRNA-seq data sets from human iNKT cells recovered from the peripheral blood (GSE128243^19^) were combined after downsampling to 600 cells (200 cells per donor, 3 donors) to match the thymic data given the very different number of cells contained in the two data sets.

## 3. Results and discussion

**Fig. 1.**
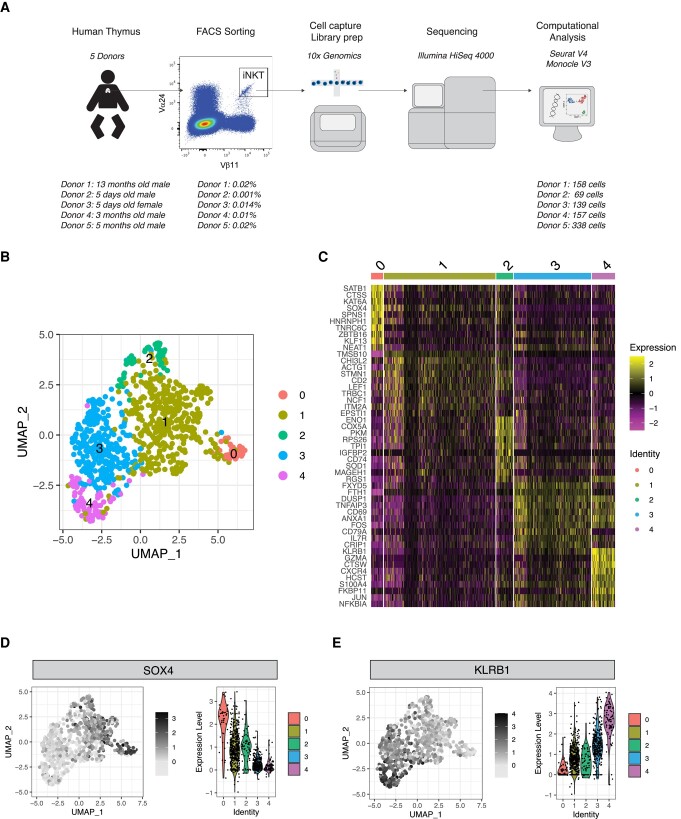
Single-cell transcriptomic analysis reveals heterogeneity of human thymic iNKT cells. (A) Schematic representation of the experimental pipeline. (B) UMAP plot of scRNA-seq data showing distinct clusters of human thymic iNKT cells. (C) Single-cell heatmap representing the 10 most highly differentially expressed genes in human thymic iNKT cell clusters. Expression for each gene is scaled (*z*-scored) across single cells. (D, E) Relative expression and normalized counts of SOX4 (D) and KLRB1 (E) in human thymic iNKT cell clusters.

**Fig. 2.**
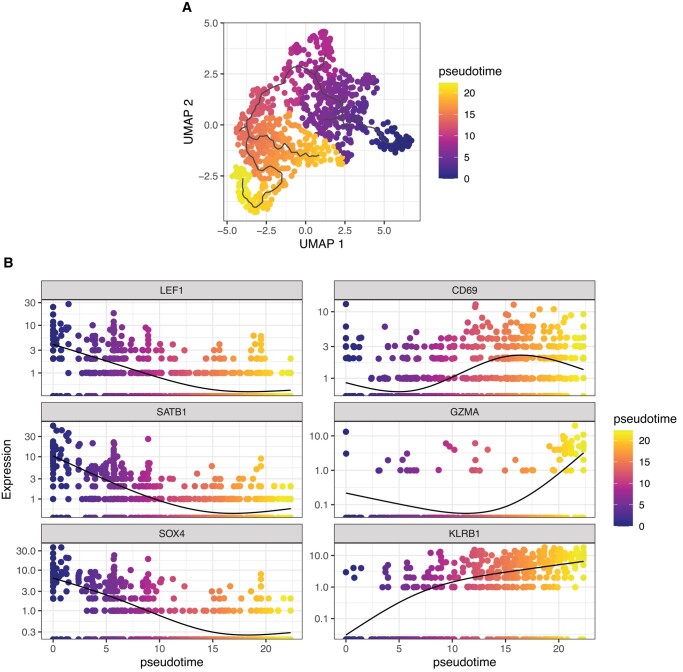
Pseudotime analysis indicates ontological relationship between human thymic iNKT cell clusters. (A) UMAP plot of scRNA-seq data colored by pseudotime. (B) Single-gene normalized expression levels across the pseudotime trajectory. Cells are colored by pseudotime.

We performed scRNA-seq on human thymic Vα24^+^Vβ11^+^ iNKT cells FACS-sorted from thymi from 5 newborn/infant donors (Fig. 1A). After removing doublets (>2,500 gene counts), cells with lowest (<100) gene counts, and cells with high (>10%) mitochondrial gene content, 862 cells were retained for downstream analysis (Fig. 1A). Unsupervised Uniform Manifold Approximation and Projection (UMAP) analysis segregated human thymic iNKT cells into 5 clusters (Fig. 1B). Such distribution was conserved across the 5 samples originating from the 5 different donors (Supplementary Fig. 1A). Cluster 0 (C0) was the smallest cluster, representing on average 4.9% of total cells (Supplementary Fig. 1B). Differential expression analysis performed to identify genes preferentially expressed in each cluster revealed that iNKT cells in C0 were enriched for genes previously reported in mice to be associated with development of iNKT cell precursors, including SRY-box transcription factor 4 (SOX4^20^) and special AT-rich sequence-binding protein 1(SATB1^21^) (Fig. 1C). Cluster 1 (C1) was the biggest one, representing on average 50.4% of all thymic iNKT cells (Supplementary Fig. 1B), and expressed other genes involved in iNKT cell development, namely, lymphoid enhancing factor 1 (LEF1^22,23^) and integral membrane protein 2A (ITM2A^9,24^) (Fig. 1C). SOX4, encoding the transcription factor important for the development of iNKT cells,^20^ was preferentially expressed in cluster 0 and, to a lesser extent, in C1 (Fig. 1D). SOX4 and LEF1 have been shown to be important transcription factors for iNKT cell development.^20^ LEF1 is a crucial transcription factor for iNKT cell proliferation,^22,23^ and two extensive studies of murine thymic iNKT cells showed that LEF1 and SOX4 are highly upregulated in NKT0 cells.^9,24^ ITM2a, a gene preferentially expressed in C1, was also highly upregulated in the NKT0 population in these analyses.^9,24^ ITM2A is a target gene of GATA3, a transcription factor involved in differentiation of murine iNKT cells, especially for the production of T_h_2 cytokines by iNKT cells.^25^ Cells in cluster 2 (C2) expressed relatively high levels of SOX4, lower than C0 and similar to C1, but expressed high levels of CD74 (Fig. 1C), a gene associated with T cell–T cell contact and involved with the class II major histocompatibility complex, which has recently been linked to the ST1 differentiation state.^26^ Cluster 3 (C3) represented the second largest cluster (on average 29.4%; Supplementary Fig. 1B) among thymic iNKT cells and expressed higher levels of genes associated with iNKT full differentiation such as killer cell lectin-like receptor B1 (KLRB1, encoding the surface molecule CD161; Fig. 1E), a molecule associated with effector function in several lymphocytic subsets^27^ (Fig. 1C). To note, C3 cells expressed higher levels of CD69 (Fig. 1C) transcript, encoding a surface molecule associated with an activated state in T cells^28^ and augmented cytotoxicity in NK cells.^29,30^ The highest levels of KLRB1 transcripts were expressed by cluster 4 (C4), a small cluster representing on average 8% of thymic iNKT cells (Supplementary Fig. 1B) that was significantly enriched in molecules involved in cytotoxic effector functions, namely, NKG7, encoding a protein essential for cytotoxic granule exocytosis in effector lymphocytes (Supplementary Fig. 2A), and GZMA and PRF1, encoding the cytotoxic effector molecules granzyme A and perforin, respectively (Supplementary Fig. 2B and C). Overall, the gene program of cells in C0 and, to a lesser extent, C1 and C2 is compatible with an earlier developmental stage with higher expression of genes involved in iNKT cell development and proliferation, whereas cells in C3 and C4 resembled murine mature iNKT cells at a later stage of differentiation, with an upregulation of molecules associated with an activated phenotype in C3 and high expression of genes responsible for effector functions in C4. Based on these data, we hypothesized that the 5 clusters could correspond to different stages of human iNKT development. To address this hypothesis, we performed a pseudotime analysis with C0 defined as the root. This analysis revealed a cell transition trajectory from C0 to C1, C3 and C4 (Fig. 2A). Along such trajectory, we observed a progressive downregulation of the genes encoding the transcription factors LEF1, SATB1, and SOX4 (Fig. 2B). This was mirrored by the upregulation of genes known to be involved in iNKT terminal differentiation (KLRB1^7,24^) and production of cytotoxic molecules (GZMA), revealing a dynamic process of cells in C0 maturing to cells in C1 and C3, resulting in a cytotoxic phenotype of the differentiated cells in C4. Taking these findings together, we suggest that different maturation stages of iNKT cells exist in human thymus. These findings are in line with previously reported murine data showing that different maturation stages of iNKT cells are present at the thymic level.^9,24^

**Fig. 3.**
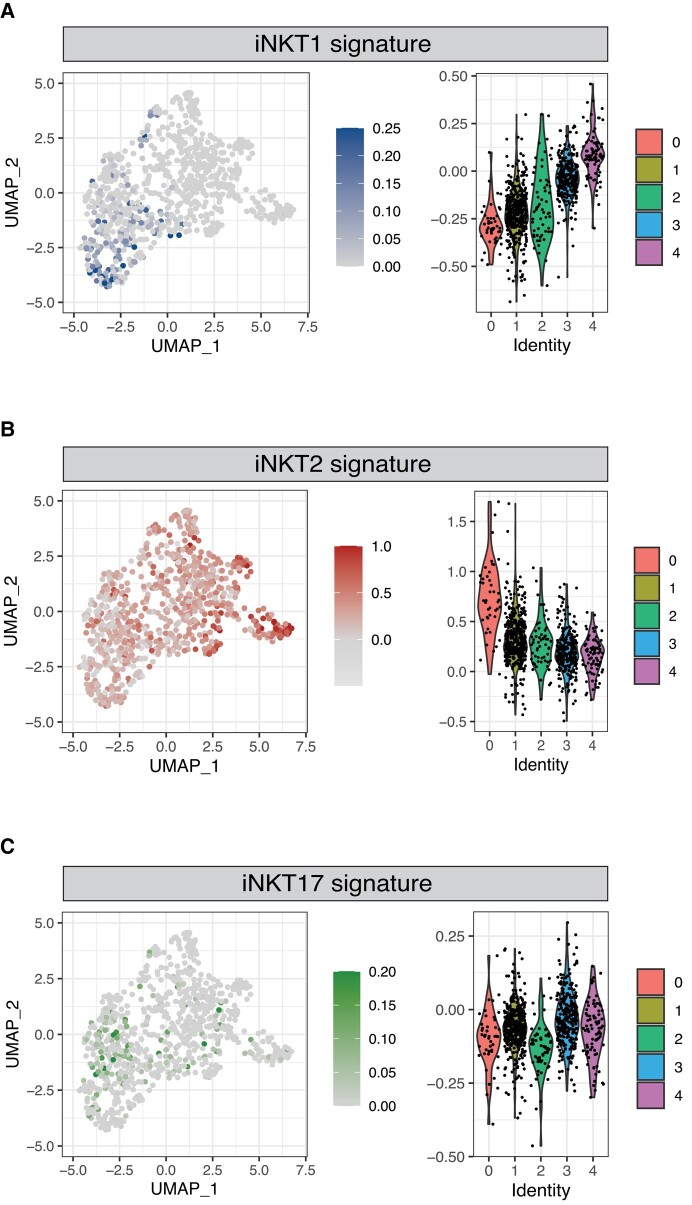
Identification of human iNKT cells expressing an iNKT1 and iNKT2 signature. Normalized expression of iNKT1 (A), iNKT2 (B), and iNKT17 (C) signatures in thymic iNKT cells. Color intensity and violin plots represent the composite expression score of iNKT sublineage-specific gene sets as calculated using Seurat's AddModuleScore function.

Murine iNKT cells have been shown to differentiate at the thymic level into 3 sublineages—namely, iNKT1, iNKT2, and iNKT17 cells—displaying different transcriptomic,^11,24^ epigenomic,^11^ and functional^13^ characteristics. We therefore investigated whether human thymic iNKT cells also displayed signs of sublineage commitment. To this aim, we generated subset-specific gene signatures for iNKT1, iNKT2, and iNKT17 based on human gene orthologs of genes we previously identified in single-cell transcriptomic analysis of murine thymic iNKT cells (Supplementary Table 1).^13^ We chose this strategy rather than looking at single markers, such as the genes encoding the transcription factors T-bet, PLZF, GATA3, and RORγt, because of the nonlinear correlation between gene transcript detected by scRNAseq and protein levels. Our analysis detected an enrichment in iNKT1 signature-expressing cells in C4 and, to a lesser extent, in C3 (Fig. 3A). Conversely, cells in C0 displayed an enrichment for iNKT2 signature-expressing cells (Fig. 3B). We did not identify any clear pattern of distribution of iNKT17 signature-expressing cells (Fig. 3C). To validate these results, we performed a similar analysis using the iNKT subset gene signatures originally reported by Engel and colleagues in their seminal study.^11^ This analysis confirmed the highest expression of the iNKT1 gene signature in C4 (Supplementary Fig. 3A). Similarly, it identified an enrichment in the iNKT2 gene signature in C0 (Supplementary Fig. 3B) and did not reveal any clearly defined expression of the iNKT17 signature across the cell subsets (Supplementary Fig. 3C). Collectively, our analysis identifies heterogeneity among thymic iNKT cells and clearly demonstrates the existence of a cluster of cells displaying a transcriptional profile corresponding to the one reported in murine iNKT1 cells. These results demonstrate for the first time, to our knowledge, that human iNKT cells undergo full differentiation in the thymus. Conversely, iNKT cells with an iNKT17 profile did not cluster in one specific region in the UMAP analysis. Thus, we cannot conclude that iNKT17 cells also exist in human thymus. As human iNKT cells that express RORγt and IL-17 have been described in peripheral blood,^31^ it is possible that they either differentiate in the periphery^32^ or emigrate directly after differentiation. In addition to these data, several studies suggest that CD69 expression of iNKT1 cells is responsible for thymic retention^24,33^ and that only iNKT2, iNKT17, and some iNKT1 subsets leave the thymus.^24^

**Fig. 4.**
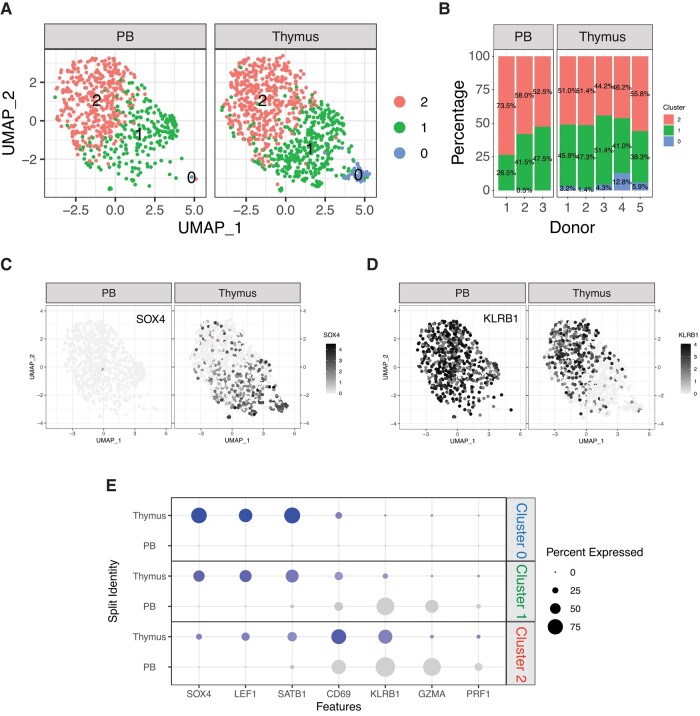
Comparison of human iNKT cells from thymus and peripheral blood. (A) UMAP plot of scRNA-seq data showing distinct clusters of human iNKT cells from thymus and peripheral blood. (B) Stacked plots indicating the proportions of different iNKT cell clusters in samples from human thymus and peripheral blood. (C, D) Relative expression and normalized counts of SOX4 (C) and KLRB1 (D) in human iNKT cell clusters depending on the tissue origin. (E) Bivariable heatmap representing the percentage (circle size) and the intensity (circle color) of expression of genes reflecting iNKT cell development and differentiation depending on the cluster and the tissue origin.

To compare the distribution of the iNKT subsets identified in the thymus to the one they display in the periphery, we next performed a combined analysis of our data set with publicly available scRNA-seq data obtained on iNKT cells isolated from human peripheral blood^19^ (Fig. 3). The combined analysis identified 3 clusters (Fig. 4A). A first cluster (cluster 0′, C0′) was observed almost exclusively in the thymus, where it represented on average 5.5% of iNKT cells, while it was virtually absent in the peripheral blood (Fig. 4B). Conversely, clusters 1′ (C1′) and 2′ (C2′) were present at similar proportions in peripheral blood and thymus (Fig. 4B). In terms of gene expression, C0′ displayed a phenotype reminiscent of the aforementioned C0, with the highest levels of SOX4 (Fig. 4C and E) as well as LEF1 and SATB1 (Fig. 4E). Conversely, C2′ displayed in both peripheral blood and thymus a more differentiated phenotype associated with expression of KLRB1 (Fig. 4D and E), CD69, and GZMA (Fig. 4E). C1′ displayed an intermediate transcriptomic profile that differed depending on the tissue of origin, SOX4^+^KLRB1^−^ in the thymus and SOX4^−^KLRB1^+^ in the peripheral blood (Fig. 4C, D, E). In conclusion, our analysis revealed the existence of a population of immature human iNKT cells exclusively present in the thymus and of a more differentiated one originating in the thymus that persists and becomes predominant in the peripheral blood.

Before iNKT subsets in mice were investigated, data suggested that iNKT cells are defined by their maturation stage.^7,34^ Later iNKT1, iNKT2, and iNKT17 cells were identified as fully differentiated cell populations.^10,35^ More recent reports performed scRNA-seq and showed with trajectory analysis that iNKT cells in the thymus differ in their maturation stage.^9,24^ Moreover, they showed that iNKT1 and iNKT2 cells consist of different subgroups and suggest a plasticity at least in iNKT0 and NKTp cells and in the more immature subgroups of iNKT2 cells.^9,24^ Thus, owing to these studies, it is now possible to reconcile the discovery of iNKT1, iNKT2, and iNKT17 subsets with the knowledge of different maturation stages of iNKT cells in the thymus, suggesting that iNKT2 cells display immature characteristics compared to iNKT1 and iNKT17 cells and probably function as a precursor for these subsets.^9,24^ Moreover, a recent study of thymic porcine iNKT cells also demonstrated that some iNKT2 cell subsets of prepubertal pigs had immature properties with an upregulation of LEF1 and SATB1 that is typical for immature iNKT cells.^14^ Intriguingly, these data are also in line with our findings that cells with an iNKT2 signature are present in the human thymus, yet appear with immature properties.

As the composition of iNKT cells is altered with aging^36^ and might differ among individuals, our analysis represents a snapshot of iNKT cell distribution at a very young age. Furthermore, the number of cells in our analysis is limited due to the paucity of iNKT cells in human thymus and the difficulties in accessing thymic tissue. This might especially limit our ability to detect minor cell subsets. We can therefore not exclude that other intermediate or fully differentiated states exist but have been missed by our analysis due to the limited number of cells.

To our knowledge, this is the first study investigating the transcriptomic differences of human thymic iNKT cells, with respect to iNKT maturation stages and cell subsets. Moreover, our data are in line with recent murine studies showing that iNKT cells with iNKT2 properties have immature characteristics and can give rise to terminally differentiated iNKT1 cells. Our work is a first step to a better understanding of human iNKT cell heterogeneity at the thymic level. Future studies will explore human iNKT subsets and decipher their function.

## Supplementary Material

qiad113_Supplementary_Data

## Data Availability

The data sets generated for this study can be found in the Gene Expression Omnibus database (accession number GSE210956).
