# Supplementary figures and images for: Single-cell transcriptomics reveal different maturation stages and sublineage commitment of human thymic invariant natural killer T cells
Source: J Leukoc Biol. 2023 Sep 23;115(2):401–9. doi: 10.1093/jleuko/qiad113 (PMC10799303; doi:10.1093/jleuko/qiad113)

SUPPLEMENTAL FIGURE 1

A

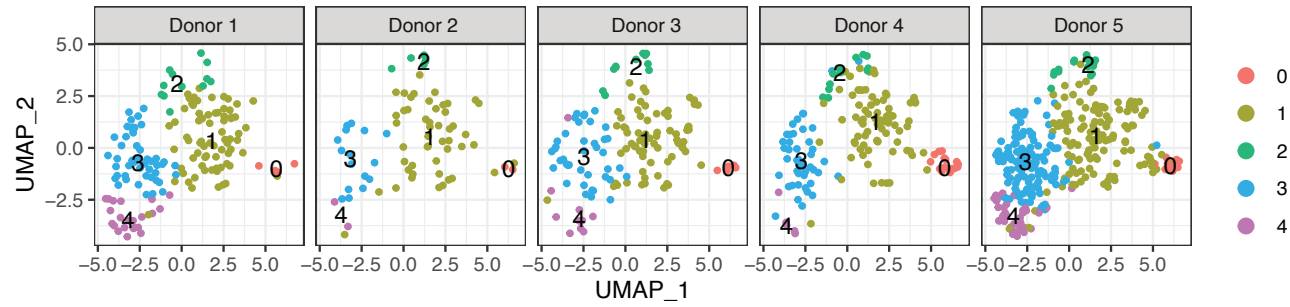

B

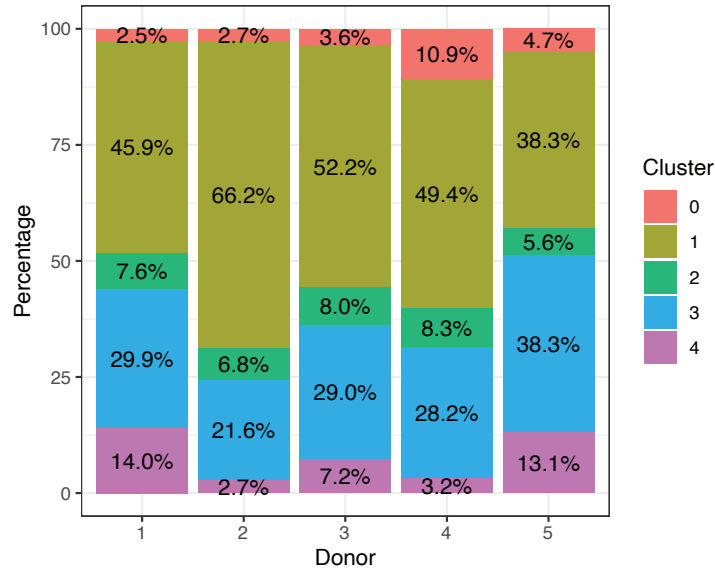

Supplement: qiad113_Supplementary_Data [file qiad113_supplementary_data.zip › MaasBauer_JLB_Revision1_SupplementalFigure1_10072023.pdf]

SUPPLEMENTAL FIGURE 2

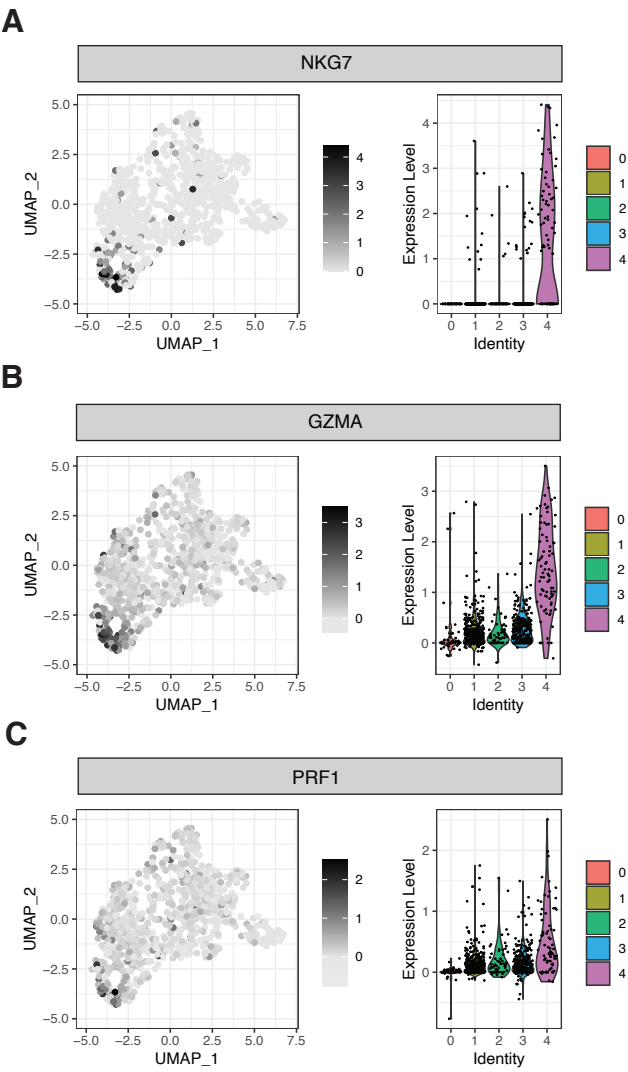

Supplement: qiad113_Supplementary_Data [file qiad113_supplementary_data.zip › MaasBauer_JLB_Revision1_SupplementalFigure2_10072023.pdf]

SUPPLEMENTAL FIGURE 3

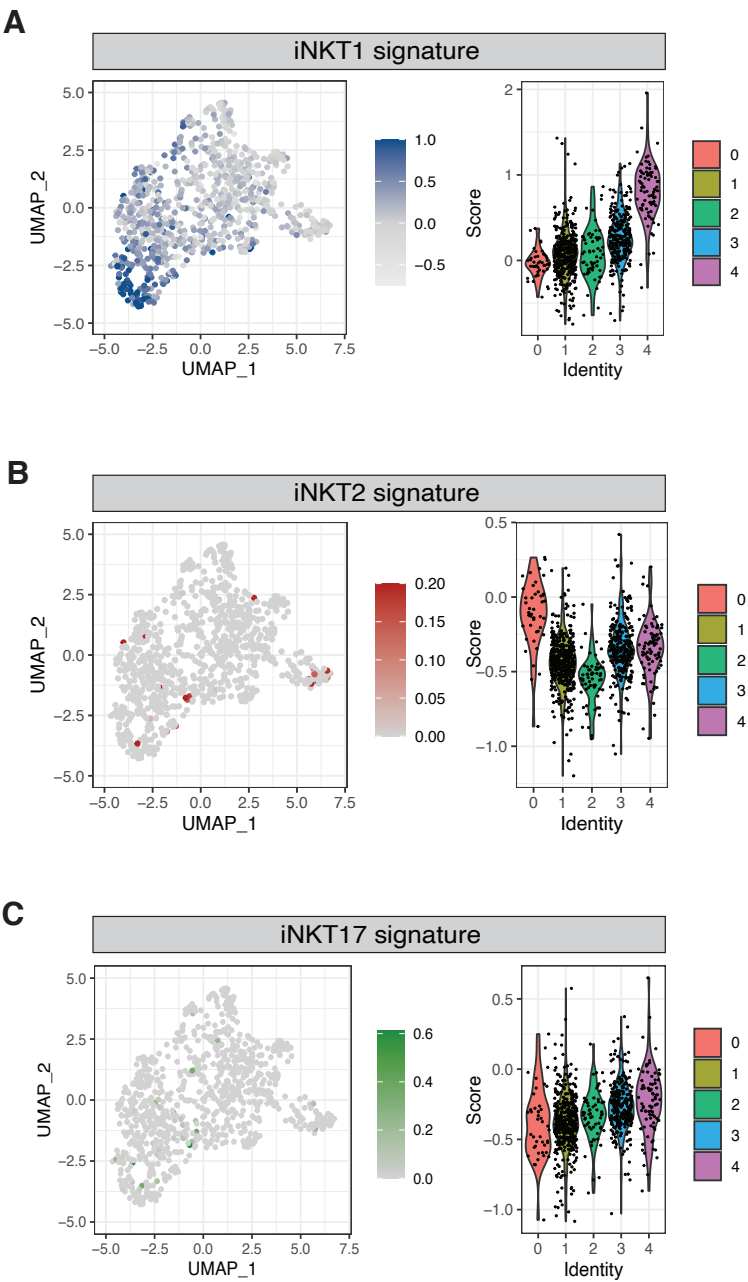

Supplement: qiad113_Supplementary_Data [file qiad113_supplementary_data.zip › MaasBauer_JLB_Revision1_SupplementalFigure3_10072023.pdf]
